# Charge Transfer in n-FeO and p-α-Fe_2_O_3_ Nanoparticles for Efficient Hydrogen and Oxygen Evolution Reaction

**DOI:** 10.3390/nano14181515

**Published:** 2024-09-18

**Authors:** Amir Humayun, Nandapriya Manivelan, Kandasamy Prabakar

**Affiliations:** Advanced Sustainable Energy Laboratory, Department of Electrical and Electronics Engineering, Pusan National University, 2 Busandaehak-ro 63beon-gil, Geumjeong-Gu, Busan 46241, Republic of Korea

**Keywords:** n-FeO, p-α-Fe_2_O_3_, HER, OER, H-cell, electrocatalyst, full cell water-splitting applications

## Abstract

This study aims to explore the n-FeO and p-α-Fe_2_O_3_ semiconductor nanoparticles in hydrogen (HER) and oxygen (OER) evolution reactions and a combined full cell electrocatalyst system to electrolyze the water. We have observed a distinct electrocatalytic performance for both HER and OER by tuning the interplay between iron oxidation states Fe^2+^ and Fe^3+^ and utilizing phase-transformed iron oxide nanoparticles (NPs). The Fe^2+^ rich n-FeO NPs exhibited superior HER performance compared to p-α-Fe_2_O_3_ and Fe(OH)_x_ NPs, which is attributed to the enhancement in n-type semiconducting nature under HER potential, facilitating the electron transfer for the reduction in H^+^ ions. In contrast, p-α-Fe_2_O_3_ NPs demonstrated excellent OER activity. An H-cell constructed using n-FeO||p-α-Fe_2_O_3_ NPs as cathode and anode achieved a cell voltage of 1.87 V at a current density of 50 mA/cm^2^. The cell exhibited remarkable stability after 30 h of activation and maintained the high current density of 100 mA/cm^2^ for 80 h with a negligible increase in cell voltage. This work highlights the semiconducting properties of n-FeO and p-α-Fe_2_O_3_ for the electrochemical water splitting system using the band bending phenomenon under the applied potential.

## 1. Introduction

Hydrogen is considered the energy of the future due to its high energy density and sustainability. It is expected to be an alternative energy source to fossil fuels with low carbon footprints. Its large-scale production could significantly impact transportation, electricity generation, and industrial processes by creating a cleaner and more secure energy future [1,2,3]. Moreover, hydrogen production through electrochemical water splitting (EWS) is environmentally friendly if renewable energy sources generate electricity. Even though the EWS system is totally dependent on surface kinetics, the higher charge-transfer resistance and low adsorption energy of H* and OH* reaction intermediates demand a higher overpotential for hydrogen and oxygen evolution reactions (HER and OER) [4,5]. Therefore, developing an efficient electrocatalyst is essential to overcome the barriers (overpotential) and accelerate these surface kinetics [6,7,8].

In recent years, earth-abundant nanostructured materials have attracted significant attention as catalysts and in energy conversion applications since they could improve the charge transfer and electrochemical reaction mechanisms [9,10,11,12,13,14,15]. The iron oxides, particularly those containing oxygen vacancies, have garnered significant attention as potential electrocatalysts for both HER and OER due to their abundance, low cost, and environmental friendliness [16,17,18,19]. The unique electronic structure of iron oxides, coupled with the presence of oxygen vacancies, offers a fertile ground for tuning catalytic properties and enhancing the overall efficiency of water splitting. Moreover, the fundamental principles underlying the HER process demand a supply of electrons for the reduction, whereas OER demands the extraction of electrons for the oxidation to occur at the electrolyte. Therefore, developing a highly active OER catalyst requires high work function, whereas HER demands low work function materials for effective electrolysis. Hence, a single material may not be used for both the dual functional HER and OER applications [20,21,22].

Moreover, it is reported that the oxygen vacancy engineering in metal oxide nanoparticles can enhance HER activity and stability by creating more active sites and promoting charge transfer [23]. The oxygen vacancies create new electronic states within the bandgap of iron oxide, leading to enhanced hydrogen adsorption and faster reaction kinetics [24]. On the other hand, α-Fe_2_O_3_ (hematite) has been used as an OER electrocatalysis due to its suitable bandgap, chemical stability, and photo-corrosion resistance [25,26,27]. However, its poor electrical conductivity and slow charge transfer kinetics often limit its OER activity. Earlier studies have shown that oxygen vacancies in α-Fe_2_O_3_ can significantly enhance OER performance. For instance, incorporating Co^2+^ into the Fe^3+^ crystal lattice of FeO(x) enhances the electrocatalytic properties of Co sites and boosts the OER performance in alkaline conditions [28]. Similarly, it has been reported that adding Fe^3+^ into the electrolyte reduces the overpotential and Tafel slope in CoO_(x)_ catalysts toward OER [29]. The Fe_2_O_3_@NiO heterojunctions with enriched oxygen vacancies in Fe_2_O_3_ NPs lead to enhanced OER activity [30].

Though many studies have reported the individual HER and OER properties of n-FeO and p-α-Fe_2_O_3_, there has been limited research on their combined performance for overall water-splitting applications. This work aims to bridge this gap by exploring the properties of n-FeO and p-α-Fe_2_O_3_ in a single full cell electrocatalyst system. By tuning the interplay between iron oxidation states Fe^2+^ and Fe^3+^ and utilizing the phase-transformed iron oxide nanoparticles (NPs), we aim to achieve distinct electrocatalytic performances for both HER and OER. We hypothesize that n-FeO NPs, enriched in Fe^2+^, will excel in HER due to their potent redox mediation capabilities, while p-α-Fe_2_O_3_ NPs, enriched with Fe^3+^, will demonstrate superior OER activity. This study will provide valuable insights into designing and developing efficient and stable iron oxide-based electrocatalysts for overall water splitting and might pave the way for sustainable hydrogen production.

## 2. Experimental Section

### 2.1. Materials

Ferric chloride hexahydrate (FeCl_3_·6H_2_O), ruthenium (IV) oxide (RuO_2_), ammonia solution, sodium hydroxide (NaOH), potassium hydroxide (KOH), carbon black (acetylene, 100%; compressed, 99.9%), N-methyl-2-pyrrolidone (NMP, ≥99.5%), and polyvinylidene fluoride (PVDF) were purchased from Sigma-Aldrich (Busan, Republic of Korea). Nickel foam (NF) was purchased from 4Science, Suwon, Republic of Korea. The H-cell, Nafion (Model: N115, Thickness: 127 mM), and film were obtained from NARA-Cell Tech Corporation, Seoul, Republic of Korea.

### 2.2. Synthesis of n-FeO

**Scheme 1:**
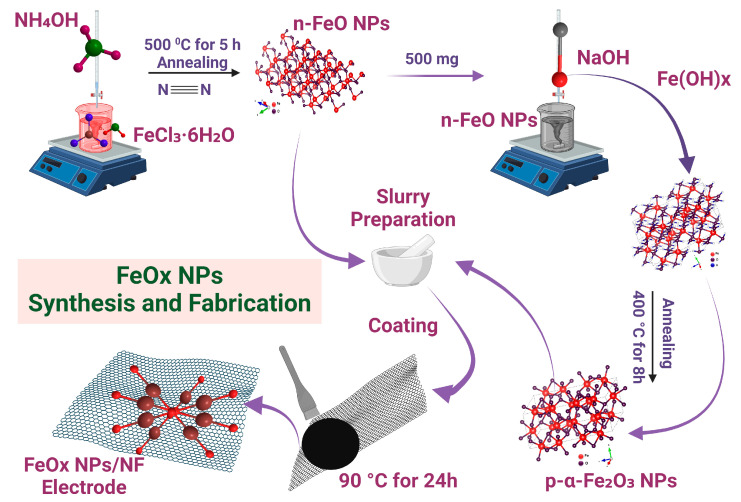
Schematic illustration of the synthesis of n-FeO, Fe(OH)_x_, and p-α-Fe_2_O_3_ nanoparticles and the electrode fabrication.

The n-FeO (Fe^2+^) nanoparticles (NPs) were synthesized using FeCl_3_·6H_2_O as a precursor. At room temperature, 4.75 g of FeCl_3_·6H_2_O was dissolved in 50 mL of deionized (DI) water under magnetic stirring. A 40 mL aqueous ammonia solution was then added dropwise to the solution. The resulting mixture was stirred continuously for 24 h, and a green precipitate was formed. The precipitate was then filtered and thoroughly washed with DI water and ethanol. Furthermore, the green residue was dried at 80 °C for 12 h in a hot air oven and subsequently annealed at 500 °C for 5 h (N_2_ atmosphere) to obtain the final n-FeO NPs (Scheme 1).

### 2.3. Synthesis of p-α-Fe_2_O_3_

The previously synthesized n-FeO NPs powder was the starting material for p-α-Fe_2_O_3_ nanoparticle synthesis. A 500 mg of n-FeO NPs was mixed with 5 g of NaOH pellets dissolved in 100 mL DI water at room temperature. The mixture was stirred until a clear solution was formed. The reaction between n-FeO and hydroxide ions (OH^−^) in the solution resulted in Fe(OH)_x_ formation. After 60 min, the settled precipitate was filtered and dried in a hot oven at 80 °C for 12 h. The dried brown particles were washed several times with DI water until the pH approached 7.0. Finally, the residue was annealed at 400 °C for 8 h and cooled slowly to room temperature. During this process, the Fe(OH)_x_ reacted with oxygen (O_2_) to form the final p-α-Fe_2_O_3_ NPs (Scheme 1).

### 2.4. Electrode Preparation

The NF (1 × 1 cm^2^ geometrical area) electrode was cleaned using ethanol sonication and dried in an oven. Afterward, 80% (4 mg) of n-FeO and p-α-Fe_2_O_3_ NPs were blended individually using 10% carbon black and 10% PVDF and grained. Subsequently, a few drops of NMP solvent were added and grained in mortar to prepare a slurry. Then, the slurry mixture was loaded on an NF electrode and dried overnight at 60 °C in an oven (Scheme 1). After drying, the n-FeO, Fe(OH)x, and p-α-Fe_2_O_3_ loaded electrodes were used in electrochemical catalytic studies.

### 2.5. H-Cell Fabrication

The H-cell electrochemical analyzer was used for the overall water-splitting process (Scheme S1). The cell was designed with two compartments containing 25 mL of 1 M KOH electrolyte solution. A Nafion membrane, pre-treated by sequential sonication and washing in 3% H_2_O_2_, 0.5 M H_2_SO_4_, ethanol−water mixture, and finally deionized water, was used to separate the anode and cathode compartments. In this alkaline environment, the sulfonic acid groups in the Nafion membrane are deprotonated, allowing for the passage of hydroxide ions (OH^−^) while hindering the transport of other ions, effectively acting as an anion exchange membrane. The n-FeO NPs serve as the cathode for the HER, while p-α-Fe_2_O_3_ NPs act as the anode for the OER. The n-FeO||p-α-Fe_2_O_3_/NF electrode was used for the overall water splitting.

### 2.6. Electrochemical Measurements

The electrochemical measurements were performed using a Biologic SP-150 Potentiostat in a three-electrode system. Hg/HgO, graphite rod, and iron oxide (FeOx) NPs deposited on NF were employed as reference, counter, and working electrodes. The experiment was performed in 1 M KOH alkaline media, and N_2_ gas was purged for 20 min before the experiment. The linear sweep voltammetry (LSV) was carried out at a scan rate of 1 mV/s with 100% iR correction. The iR correction was made before LSV measurements to avoid ohmic loss (iR loss) using E = E_(RHE)_ − iR, where E is the iR corrected potential concerning the RHE, i is the measured current, and R is the uncompensated resistance calculated from Electrochemical Impedance Spectroscopy (EIS). The LSV was obtained at a low scan rate to minimize the pull current.

## 3. Results and Discussion

**Figure 1:**
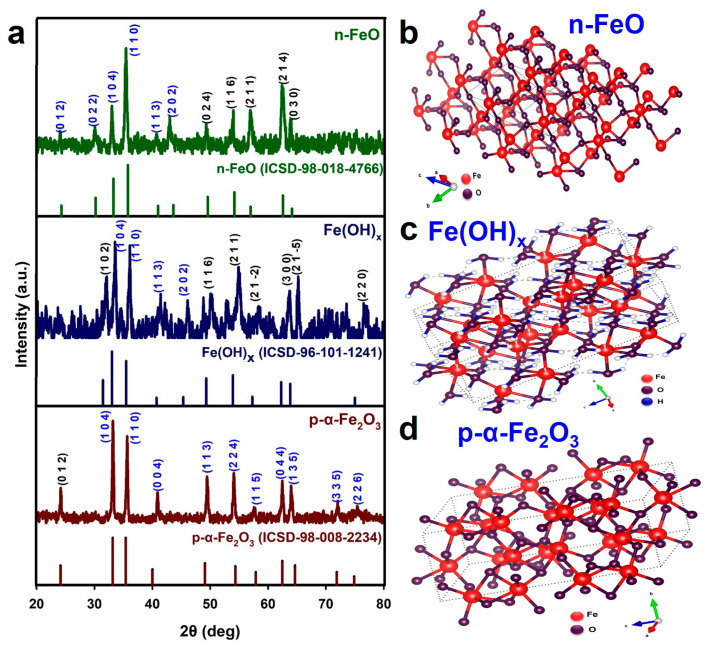
(**a**) XRD diffraction patterns of n-FeO, Fe(OH)_x_, and p-α-Fe_2_O_3_ nanoparticles. The crystal structures of (**b**) n-FeO, (**c**) Fe(OH)_x_, and (**d**) p-α-Fe_2_O_3_ were drawn using VESTA software 3.5.8 @2022.

The synthesized FeOx NPs and their crystalline properties were analyzed using X-ray diffraction (XRD). The n-FeO NPs exhibited a monoclinic crystal structure with lattice parameters a = 4.94 Å, b = 5.40 Å, and c = 7.46 Å, as confirmed by the XRD data and is matched with ICSD card #98-018-4766 (Figure 1a). The observed 2θ values were found at 24.00°, 30.05°, 32.96°, 35.49°, 40.67°, 42.98°, 49.35°, 54.07°, 56.86°, 62.51°, and 63.75° and their corresponding hkl indices ((0 1 2), (0 2 2), (1 0 4), (1 1 0), (1 1 3), (2 0 2), (0 2 4), (1 1 6), (2 1 1), (2 1 4), and (0 3 0)), respectively [31]. This structure features the iron atoms occupying the corner and face positions, whereas O atoms occupy the unit cell’s edge and central positions (Figure 1b). The uniform distribution of Fe and O atoms throughout the crystal lattice minimizes defects and promotes structural stability, which is crucial for long-term performance [32]. The reaction between n-FeO and OH^−^ resulted in the formation of Fe(OH)_x_ during the synthesis of p-α-Fe_2_O_3_ NPs, and its structural analysis is shown in Figure 1c. The observed 2θ values (31.45°, 33.18°, 35.59°, 40.65°, 45.13°, 49.26°, 53.75°, 56.96°, 62.25°, 63.74°, and 74.88°) and their corresponding hkl indices ((1 0 2), (1 0 4), (1 1 0), (1 1 3), (2 0 2), (1 1 6), (2 1 1), (2 1 -2), (3 0 0), (2 1 -3), and (2 2 0)), respectively are consistent with ICSD card #96-101-1241, confirming the formation of Fe(OH)_x_ NPs in the hexagonal crystal structure where Fe-O-H are arranged in the hexagonal unit cell [33]. The p-α-Fe_2_O_3_ NPs were prepared by calcining the Fe(OH)_x_ NPs, which adopted a rhombohedral crystal structure, as shown in Figure 1d. The observed 2θ values (24.12°, 33.18°, 35.66°, 40.88°, 49.33°, 54.01°, 57.44°, 62.40°, 64.02°, 72.10°, and 75.50°) and their corresponding hkl indices ((0 1 2), (1 0 4), (1 1 3), (0 0 4), (1 3 3), (2 2 4), (1 1 5), (0 4 4), (1 3 5), (3 3 5), and (2 2 6)), respectively matched with ICSD card #98-008-22349 [34]. The grain size was 59.8 nm in p-α-Fe_2_O_3_ NPs and is highly crystalline compared to n-FeO and Fe(OH)_x_ NPs.

**Figure 2:**
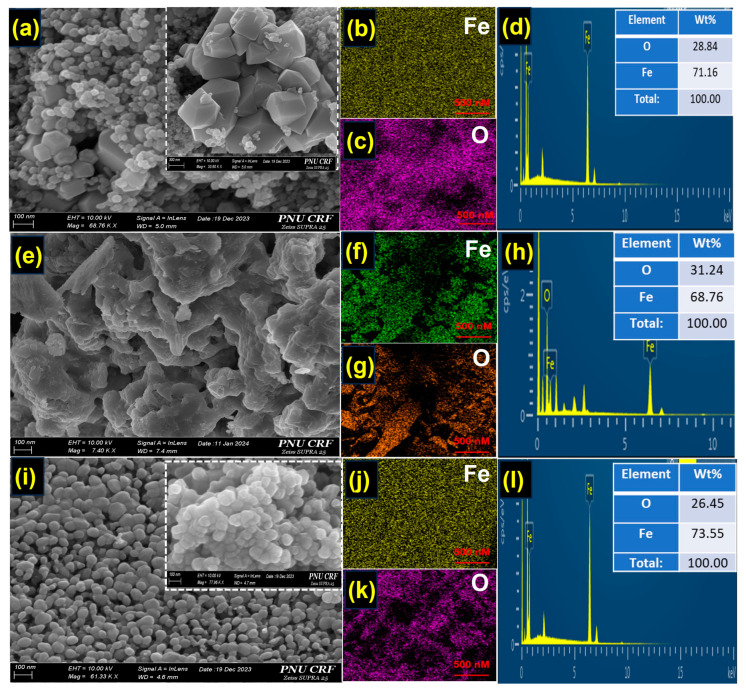
FE-SEM morphologies of (**a**) n-FeO, (**e**) Fe(OH)_x_, and (**i**) p-α-Fe_2_O_3_ NPs; Elemental mapping of Fe and O elements in (**b**,**c**) n-FeO, (**f**,**g**) Fe(OH)_x_, and (**j**,**k**) p-α-Fe_2_O_3_ NPs; EDX analysis for (**d**) n-FeO, (**h**) Fe(OH)_x_, and (**l**) p-α-Fe_2_O_3_ NPs.

The FE-SEM images (Figure 2a,e,i) reveal that the n-FeO and p-α-Fe_2_O_3_ NPs have nanocubic crystal morphology and agree with the XRD observation. This is likely because the Fe(OH)_x_ NPs are partially amorphous due to the presence of OH-, which promote aggregation during synthesis [35]. The calcination process converts the Fe(OH)_x_ NPs to p-α-Fe_2_O_3_, resulting in a more crystalline structure due to the removal of water molecules. The EDX analysis (Figure 2d,h,l) and elemental mappings (Figure 2b,c,f,g,j,k) confirm the presence of only iron (Fe) and oxygen (O) on the surface of the NPs, indicating the purity of the prepared NPs.

**Figure 3:**
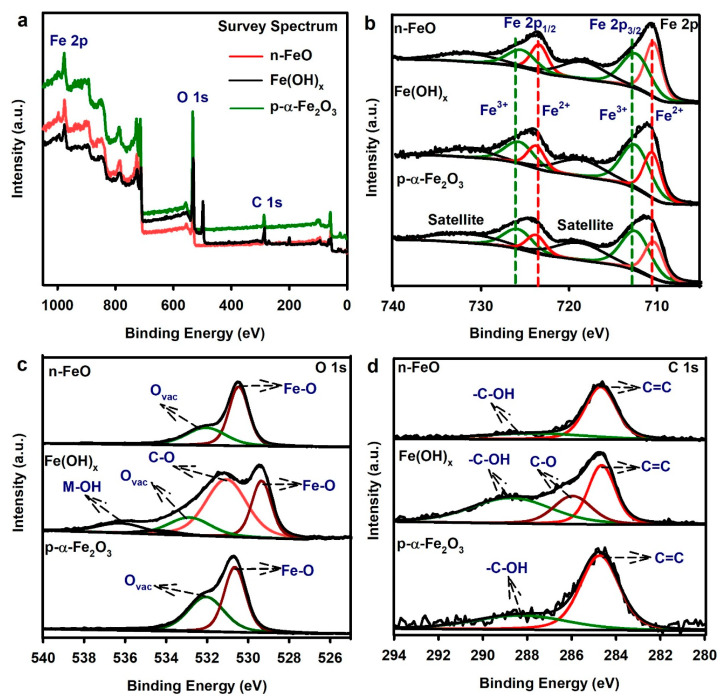
XPS analysis of n-FeO, Fe(OH)_x_, and p-α-Fe_2_O_3_ NPs, (**a**) survey spectrum, (**b**) core-level XPS spectra of Fe 2p, (**c**) core-level XPS spectra of O 1s, and (**d**) core-level XPS spectra of C 1s.

The XPS analysis (Figure 3a) reveals the surface elemental composition and oxidation states of n-FeO, Fe(OH)_x_, and p-α-Fe_2_O_3_ NPs. The survey spectrum confirms the presence of iron (Fe), oxygen (O), and adventitious carbon (C) in both samples. The high-resolution Fe 2p core-level spectra (Figure 3b) provide more detailed information about the Fe oxidation states. In all three samples, the Fe 2p spectrum exhibits peaks corresponding to both Fe^2+^ (at binding energies of 710.9 eV and 724.3 eV) and Fe^3+^ (at 712.9 eV and 726.3 eV), arising from the 2p_3_/_2_ and 2p_1_/_2_ spin-orbit splitting. Notably, the n-FeO NPs show a higher intensity for the Fe^2+^ peaks than the Fe^3+^ peaks. In contrast, the p-α-Fe_2_O_3_ and Fe(OH)_x_ NPs exhibit a more pronounced Fe^3+^ signal, indicating a higher concentration of the Fe^3+^ oxidation state in these materials [36]. The O 1s core-level spectra (Figure 3c) provide insights into the oxygen bonding environment within the materials. The peaks observed at 530.3 eV and 530.5 eV correspond to the metal-oxygen (Fe-O) bonds in the iron oxide NPs, while the peaks at 532.1 eV and 532.4 eV are attributed to oxygen vacancies (O_vac_) in these NPs [36,37]. The presence of oxygen vacancies can influence the electronic structure of iron oxides, potentially leading to enhanced catalytic activity for the OER [24]. Additionally, the peaks observed at 531.3 eV and 536.56 eV in Fe(OH)ₓ NPs are assigned to C-O and M-OH bonds, respectively. These peaks confirm the presence of hydroxide species in the samples. The C-O peak likely originates from surface contaminants, potentially arising from the organic compounds used during synthesis. The C 1s core-level spectra of both n-FeO and p-α-Fe_2_O_3_ (Figure 3d) show peaks around 284.6 eV and 288.5 eV, respectively, corresponding to adventitious carbon (C=C) and hydroxyl groups (C-OH). The C-OH bonds likely originate from organic molecules used during the synthesis of the nanoparticles.

The semiconducting nature of the synthesized iron oxide nanoparticles was investigated by the Hall effect measurements and Mott−Schottky analysis. The Hall effect measurements, conducted at room temperature under a 0.5 T magnetic field, confirmed the p-type nature of α-Fe_2_O_3_ (p-α-Fe_2_O_3_) and the n-type nature of FeO (n-FeO), with carrier concentrations of p = 1.78 × 10^10^ cm^−2^ and n = −1.760 × 10^13^ cm^−2^, respectively. Moreover, the Mott−Schottky plots [38] (Figure S1) obtained at 3 kHz exhibited negative slopes for p-α-Fe_2_O_3_ and Fe(OH)_x_, indicating the p-type behavior, while n-FeO displayed a positive slope, confirming its n-type nature [39,40,41].

### 3.1. Oxygen Evolution Reaction (OER)

**Figure 4:**
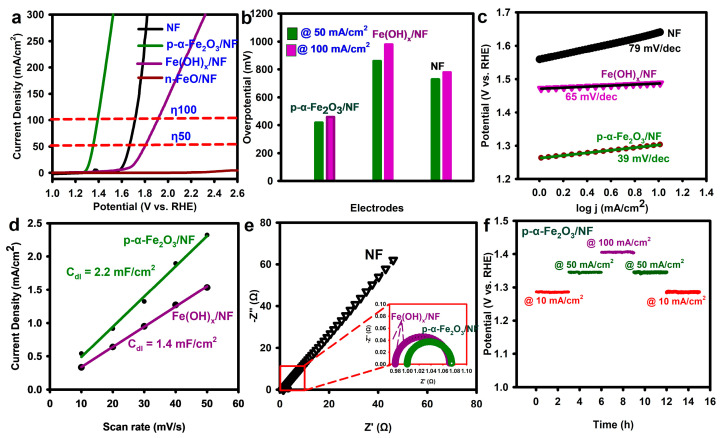
OER electrochemical studies. (**a**) LSV polarization curve, (**b**) OER overpotential, (**c**) OER Tafel slope, (**d**) Double-layer capacitance (C_dl_) slope, (**e**) EIS Nyquist plot, (**f**) Chronopotentiometry test.

The electrocatalytic performance of the prepared catalysts was measured using a three-electrode system in 1 M KOH electrolyte. All polarization curves were 100% iR-corrected. Figure 4a shows the LSV curves for the OER. The bare NF electrode exhibited onset potentials of 1.66 V and 1.71 V vs. RHE at current densities of 50 mA/cm^2^ and 100 mA/cm^2^, respectively. The p-α-Fe_2_O_3_/NF electrode demonstrated the lowest onset potentials of 1.35 V and 1.39 V vs. RHE at 50 mA/cm^2^ and 100 mA/cm^2^, respectively, outperforming the commercially available RuO_2_/NF electrode (onset potential: 1.51 V, Figure S2). The Fe(OH)_x_/NF electrode showed onset potentials of 1.79 V and 1.91 V vs. RHE at the same current densities. The n-FeO/NF electrode exhibited the lowest OER activity, possibly attributed to surface defects in the n-FeO NPs [42]. The overpotentials at different current densities are given in Figure 4b. The overpotentials for p-α-Fe_2_O_3_/NF and Fe(OH)_x_/NF electrodes to reach the current densities of 50 and 100 mA/cm^2^ were 420 and 460 mV and 860 and 980 mV, respectively. The p-α-Fe_2_O_3_ NPs oxygen vacancies help to adsorb and desorb the intermediates at the metal sites. Further, the delocalized electrons from the O^-^ go to the metal center and increase the metal’s electron density, reducing the electronegativity and the bond length between metal sites and intermediates and increasing the O_2_ evolution rate [43]. The Tafel slope in Figure 4c reflects the rate at which the current density changes with the applied potential. A lower Tafel slope indicates faster OER kinetics [44]. In this case, the p-α-Fe_2_O_3_/NF electrode demonstrates a significantly lower Tafel slope (~39 mV dec^−1^) compared to the other electrodes (65 mV/dec, and 79 mV/dec for Fe(OH)_x_/NF and bare NF, respectively). The lower Tafel slope in p-α-Fe_2_O_3_/NF suggests a faster and more favorable kinetic process for OER on this electrode. Moreover, p-α-Fe_2_O_3_ is a p-type semiconductor measured from the Hall effect measurement setup. Under OER conditions, the band bends upwards due to the p-type character, forming a positive surface charge region (enrichment layer), leaving behind a region depleted with negative charges. This band bending and positive charge region is very helpful in attracting electrons from the electrolytes and converting the OH^−^ ions into OH* intermediates, enhancing the oxidizing ability and facilitating the multi-step OER process. Further, the exact pathway for OER on p-α-Fe_2_O_3_ is still under debate, but it is generally believed that a series of single-electron transfer steps involve lattice oxygen and OH^−^ ions [45,46]. A simplified representation is given below [47].

Step 1: OH^−^ adsorbs onto a surface Fe site (denoted as *): OH^−^ + * -> *OH

Steps 2: Subsequent single electron transfers and water molecule interactions lead to the formation of an O-O bond and the release of a water molecule:

*OH -> *O + H_2_O, *O + *OH -> *OOH + *, *OOH -> O_2_ + * + H⁺

The OER mechanism at the interface between p-α-Fe_2_O_3_ and KOH electrolyte is a complex interplay between its semiconducting properties, mobile charge carriers (holes and OH^−^), and material characteristics like crystallinity and morphology. The electrochemical double-layer capacitance (C_dl_) measurements were performed in the non-Faradaic region at various scan rates (10 to 50 mV/s) to assess the electrochemical surface-active area (ECSA) of the electrodes, which reflects the number of active sites involved in OER (Figure 4d and Figure S3). The p-α-Fe_2_O_3_/NF electrode exhibits the highest C_dl_ value of 2.2 mF/cm^2^, indicating a higher active surface area compared to Fe(OH)_x_/NF (1.4 mF/cm^2^) electrode. The higher turn-over frequency (TOF) value (0.041 S^−1^) further confirms the superior OER activity of the p-α-Fe_2_O_3_/NF electrode at an overpotential of 420 mV @50 mA/cm^2^.

The Nyquist plots in Figure 4e depict the EIS used to probe the charge transfer processes at the electrode/electrolyte interface. The size of the semicircle in these plots is directly related to the charge transfer resistance (Rct) at the interface. The p-α-Fe_2_O_3_/NF electrode exhibits a significantly lower Rct value of 1.7 Ω lower than Fe(OH)_x_/NF and n-FeO/NF electrodes, indicating a faster and more efficient transfer of electrons between the electrode and the electrolyte during OER and this observation aligns well with the lower Tafel slope observed in Figure 4c [48]. The EIS measurements at positive potentials (Figure S4) further reduce the R_ct_ value in p-α-Fe_2_O_3_ and Fe(OH)_x_. This suggests that a positive bias enhances the charge transfer kinetics at the electrode surface, improving OER activity at higher operating potentials. In contrast, the Rct value increases for the n-FeO/NF electrode under positive potential. This indicates that the n-FeO electrode becomes even less favorable for OER due to a rise in the charge transfer resistance. The strong correlation between the EIS measurements (R_ct_) and the OER activities observed in Figure 4 highlights the critical role of fast charge transfer kinetics at the electrode/electrolyte interface for efficient OER electrocatalysis.

The chronopotentiometry method was employed to assess the repeatability of the p-α-Fe_2_O_3_/NF electrode (Figure 4f) under varying current densities (10 mA/cm^2^, 50 mA/cm^2^, and 100 mA/cm^2^) over a three-hour interval between the current densities. Despite the changes in the current densities, the potentiometry curve remained unchanged, indicating the high stability and repeatability of the p-α-Fe_2_O_3_/NF electrode in the KOH electrolyte.

### 3.2. Hydrogen Evolution Reaction (HER)

**Figure 5:**
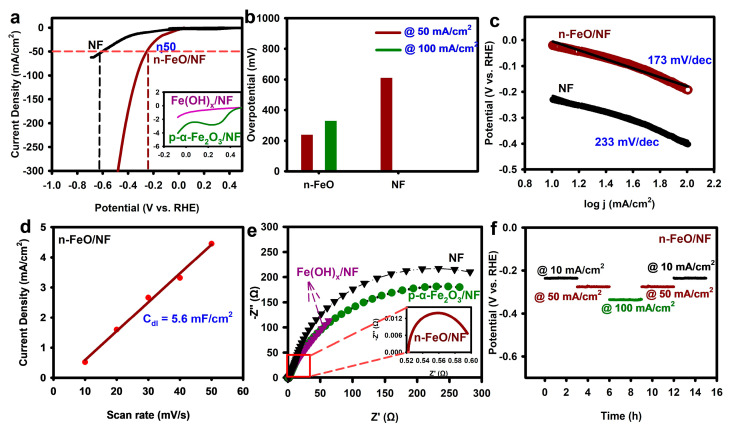
HER electrochemical studies. (**a**) LSV polarization curve, (**b**) HER overpotential, (**c**) HER Tafel slope, (**d**) Double-layer capacitance (C_dl_) slope, (**e**) EIS Nyquist plot, (**f**) Chronopotentiometry test.

The HER performance of n-FeO/NF, p-α-Fe_2_O_3_/NF, Fe(OH)_x_/NF, and bare NF electrodes was evaluated using LSV, as shown in Figure 5a. A low scan rate of 1 mV/s and a potential window of 0 to −1 V vs. RHE were employed in 1 M KOH electrolyte. The inset of Figure 5a demonstrates that p-α-Fe_2_O_3_/NF and Fe(OH)_x_/NF electrodes exhibit poor HER activity, likely due to the presence of OH^−^ ions within their structures, which can interact with the KOH electrolyte and promote charge storage behavior via K⁺ adsorption instead of facilitating the HER. In contrast, the n-FeO/NF electrode displays superior HER performance with lower onset potentials of −0.24 V and −0.33 V at current densities of 50 mA/cm^2^ and 100 mA/cm^2^, respectively. n-FeO/NF outperforms the even commercially available Pt/NF electrode (onset potential: −1.12 V, Figure S5), while the bare NF electrode exhibits an onset potential of −0.60 V at 50 mA/cm^2^. The overpotentials at current densities of −50 and −100 mA/cm^2^ are given in Figure 5b. The n-FeO/NF electrode shows small overpotentials of 250 mV and 340 mV at a −50 mA/cm^2^ and −100 mA/cm^2^ current density. The Tafel slope in Figure 5c further confirms the faster HER kinetics on the n-FeO/NF electrode. A lower Tafel slope indicates a more favorable reaction pathway. In this case, the n-FeO/NF electrode exhibits a Tafel slope of ~173 mV dec^−1^, considerably lower than the bare NF electrode (~233 mV dec^−1^). The Hall effect measurements confirm that n-FeO is an n-type semiconductor. Its bands bend downwards at the surface due to low work function, forming an electron enrichment layer when a negative potential is applied. Thus, the band banding uplifts the n-type behavior and feasibly transfers the electron to the electrolyte to reduce H^+^ ions [49,50].

The C_dl_ measurements were performed in the non-Faradaic region at various scan rates (10 to 50 mV/s) to assess the ECSA of the electrodes (Figure 5d and Figure S6). The n-FeO/NF electrode exhibits the C_dl_ value of 5.6 mF/cm^2^. The TOF value 0.034 S^−1^ observed for n-FeO NPs at an overpotential of 250 mV further validates its superior catalytic behavior for HER. Due to oxygen vacancy, the positive H^+^ ions get easily attracted in the trap and boost the reduction rate, thus reflected in increased surface area. Overall, the LSV results, overpotential, Tafel slopes, and TOF values support the n-FeO/NF as a promising HER electrode material due to its faster reaction kinetics and higher activity compared to p-α-Fe_2_O_3_/NF, Fe(OH)_x_/NF, and bare NF electrodes. Figure 5e shows the Nyquist plots in which the n-FeO NPs electrode exhibits a significantly lower Rct value of 0.7 Ω than p-α-Fe_2_O_3_/NF and Fe(OH)_x_/NF electrodes, indicating a faster and more efficient transfer of electrons between the electrode and the electrolyte during HER. The EIS measurements at various potentials (Figure S7) reveal that the n-FeO/NF electrode’s R_ct_ value decreases as the applied potential becomes more negative. This suggests that n-FeO becomes more active for HER as the negative potential increases. In contrast to n-FeO/NF, the R_ct_ values for p-α-Fe_2_O_3_/NF and Fe(OH)_x_ electrodes increase with increasing negative potential. Thus, the Fe-O structure improves the charge transfer reaction within the crystal structure by facilitating the diffusion of electrolyte ions and water molecules toward the active sites, leading to efficient mass transport during the HER process. The EIS measurements (R_ct_) correlate well with the HER activities observed in Figure 5a. These findings agree with the lower Tafel slope and higher HER activity observed for n-FeO compared to p-α-Fe_2_O_3_/NF and Fe(OH)_x_ electrodes.

The chronopotentiometry method was employed to assess the stability and repeatability of the n-FeO/NF electrode (Figure 5f) under varying current densities (10 mA/cm^2^, 50 mA/cm^2^, and 100 mA/cm^2^) over a three-hour interval between different current densities. The potentiometric curve remained unchanged, indicating the high stability and repeatability of the n-FeO/NF electrode in the KOH electrolyte.

### 3.3. Overall Water Splitting

**Figure 6:**
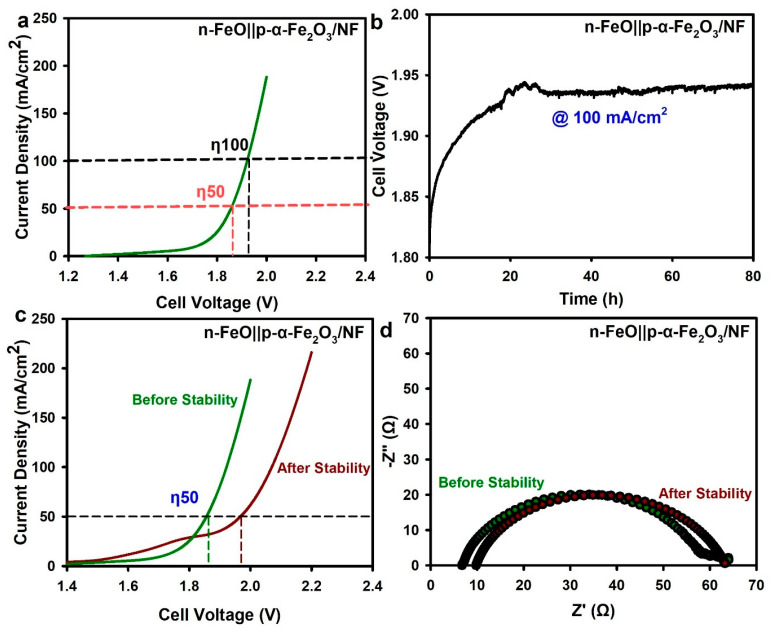
(**a**) Overall water splitting polarization curve, (**b**) Chronopotentiometry stability measurement for n-FeO||p-α-Fe_2_O_3_/NF electrode at 100 mA/cm^2^, (**c**) polarization curve before and after 80 h of stability testing, and (**d**) EIS measurement before and after 80 h of stability testing.

The assembled full cell (H-cell; Scheme S1) employed n-FeO||p-α-Fe_2_O_3_ as the electrodes for overall water splitting in 1 M KOH electrolyte. The full cell n-FeO||p-α-Fe_2_O_3_ electrodes show superior performance by exhibiting a cell voltage of 1.87 V to attain a 50 mA/cm^2^ current density (Figure 6a). Figure 6b presents the chronopotentiometric stability test of the n-FeO||p-α-Fe_2_O_3_ electrode under a constant current density of 100 mA/cm^2^. Initially, during the first 28 h of operation, a gradual increase in cell voltage from 1.87 V to 1.93 V was observed due to the electrochemical activation process, where the n-FeO cathode and p-α-Fe_2_O_3_ anode undergo surface modifications and interfacial restructuring upon interaction with the KOH electrolyte [51]. Subsequently, the cell voltage stabilizes at 1.93 V for the remaining 52 h, demonstrating the excellent long-term stability and robust electrocatalytic performance of the n-FeO||p-α-Fe_2_O_3_ electrode for water splitting. After the stability test, the cell voltage slightly increased from 1.87 V to 1.96 V@ 50 mA/cm^2^ (Figure 6c) due to the increased R_ct_ value from 58 Ω to 61 Ω as observed in the EIS analysis.

The stability-tested n-FeO and p-α-Fe_2_O_3_ electrodes were further investigated for their structural and surface analyses. The SEM images in Figure S8 show that the n-FeO electrode exhibits minimal morphological changes after the stability test, whereas the p-α-Fe_2_O_3_ (Figure S9) electrode shows significant morphological changes. This suggests that the observed morphological changes in p-α-Fe_2_O_3_ may be caused by the absorption of OH^−^ ions at the surface, which can lead to the performance degradation of the p-α-Fe_2_O_3_ electrode. This is supported by the EDX analysis, which shows a change in the Fe:O ratio after the stability test. The O content increases due to the interaction with OH^−^ ions. The stability-tested samples’ XRD results in Figure S10 imply that the n-FeO electrode structure remains unchanged, whereas the p-α-Fe_2_O_3_ electrode exhibits a peak shift in its XRD pattern. The changes in the interatomic distances may cause this shift due to the interaction with OH^−^ ions. Therefore, it is inferred from these observations that the surface of the p-α-Fe_2_O_3_ electrode should be protected, or a heterostructure can be used to extend the electrodes’ stability.

The Raman analysis of the as-deposited and stability-tested n-FeO/NF (Figure S11a) electrodes reveal three broad bands around 219, 288, and 395 cm^−1^, characteristic of n-FeO lattice vibrations. The metal-oxide bond, initially observed around 600 cm^−1^, shifts to a lower wavenumber (585 cm^−1^) with reduced intensity after the stability test. This suggests minor structural changes in the n-FeO lattice, likely due to interaction with the KOH electrolyte. However, the overall stability of the n-FeO Raman peaks indicates its good structural integrity during electrochemical operation. In contrast, the Raman spectrum of the p-α-Fe_2_O_3_/NF electrode after the stability test (Figure S11b) shows the emergence of a new peak around 983 cm^−1^. This peak is indicative of the magnetite (α-Fe_3_O_4_) phase, which undergoes partial transformation to hematite p-α-Fe_2_O_3_ upon prolonged exposure to the KOH electrolyte [52]. This transformation could be attributed to changes in the oxidation state from Fe^3+^ to Fe^2+^ under the operating conditions.

## 4. Conclusions

We have successfully synthesized n-FeO and p-α-Fe_2_O_3_ NPs and investigated their electrocatalytic activity for HER and OER in 1 M KOH electrolyte. The n-FeO NPs demonstrate promising HER performance with a low onset potential, high current density, and favorable Tafel slope. The n-type semiconducting nature of n-FeO promotes efficient electron transfer for HER. In contrast, p-α-Fe_2_O_3_ NPs exhibit superior OER activity due to their semiconducting properties, fast charge transfer kinetics, and abundant surface hydroxyl groups. The assembled n-FeO||p-α-Fe_2_O_3_ NPs-based water splitting device achieved a current density of 100 mA/cm^2^ at a cell voltage of 1.93 V and maintained stable operation for over 80 h. This work has broader implications for sustainable energy research as cost-effective water-splitting electrocatalysts. Our findings underscore the critical role of understanding the iron oxidation states, oxygen vacancies, and semiconducting behavior in the design of high-performance electrocatalysts. This knowledge can guide the development of novel materials for water splitting and various energy conversion and storage applications. By leveraging earth-abundant materials like iron oxides, we can reduce our reliance on precious metal catalysts, making sustainable energy technologies more accessible and economically viable. This study represents a significant step toward the future of renewable energy sources.

## Data Availability

Data are contained within the article.

## Supplementary Materials

The following supporting information can be downloaded at: https://www.mdpi.com/article/10.3390/nano14181515/s1, Scheme S1: H-cell fabrication; Figure S1: Mott-Schottky analysis for (a) p-α-Fe_2_O_3_/NF and Fe(OH)_x_/NF and (b) n-FeO/NF electrodes; Figure S2: OER electrochemical studies for p-α-Fe_2_O_3_/NF with commercial RuO_2_/NF LSV curve; Figure S3: Double-layer capacitance measurements for determining the electrochemically active surface area for the (a) p-α-Fe_2_O_3_/NF and (b) Fe(OH)_x_/NF electrodes in 1 M KOH, CV were measured in the non-faradaic region scan rates, varying from 10 to 50 mV/s; Figure S4: EIS measurement for (a) p-α-Fe_2_O_3_/NF, (b) Fe(OH)_x_/NF, and (c) n-FeO/NF. All are performed by positive potential regions; Figure S5: HER electrochemical studies for n-FeO/NF with commercial Pt/NF LSV curve; Figure S6: Double-layer capacitance measurements determined the electrochemically active surface area for the n-FeO/NF electrode in 1 M KOH. CV was measured in the non-faradaic region scan rates, varying from 10 to 50 mV/s; Figure S7: EIS measurement for (a) n-FeO/NF, (b) p-α-Fe_2_O_3_/NF, and (c) Fe(OH)_x_/NF. All are performed by negative potential regions; Figure S8: Post characterization FE-SEM and EDX analysis of n-FeO/NF electrode. (a and b) before stability, and (c and d) after stability; Figure S9: Post characterization FE-SEM and EDX analysis of p-α-Fe_2_O_3_/NF electrode. (a and b) before stability, and (c and d) after stability; Figure S10: Post-characterization XRD analysis of (a and b) n-FeO/NF and (c and d) p-α-Fe_2_O_3_/NF electrodes; Figure S11: Post-characterization RAMAN analysis of (a) n-FeO/NF and (b) p-α-Fe_2_O_3_/NF electrodes; Table S1: Iron Oxide NPs based catalysts for OER and HER activity. References [53,54,55,56,57,58] are included in the supplementary materials.
