# Supplementary material for: Charge Transfer in n-FeO and p-α-Fe2O3 Nanoparticles for Efficient Hydrogen and Oxygen Evolution Reaction
Source: Nanomaterials (Basel). 2024 Sep 18;14(18):1515. doi: 10.3390/nano14181515 (PMC11434590; doi:10.3390/nano14181515)
Supplement: Supplementary file 1 [file nanomaterials-14-01515-s001.zip › nanomaterials-3171798-supplementary.pdf]

<u>**Supporting Information**</u>

Charge Transfer in n-FeO and p-αFe_2O_3 Nanoparticles for Efficient Hydrogen and Oxygen Evolution Reaction

Amir Humayun, Nandapriya Manivelan, Kandasamy Prabakar*

Department of Electrical and Electronics Engineering, Advanced Sustainable Energy Laboratory, Pusan National University, 2 Busandaehak-ro 63beon-gil, Geumjeong-Gu, Busan, 46241, Republic of Korea

Electrocatalytic Calculations

All potentials used here were converted to the RHE scale using the Nernst equation. (eq S1).[1]

$$E_{RHE} = E_{Hg/HgO} + (0.0591 \times pH) + 0.098 \quad \text{........(S1)}$$

$E_{Hg/HgO}$ is the measured voltage, and the pH of 1 M KOH is 14.

The Tafel slope is a significant kinetic parameter that reflects the relationship between the electrochemical rate and overpotential, which is the potential required to achieve every 10 mA/cm^2 current density increment. It was calculated from LSV values by plotting the log of current density (j) to the overpotential (η). The Tafel equation can be obtained from the linear fitting of the Tafel curve (eq S2). [1]

$$\eta = b \log j + a \quad \text{...............(S2)}$$

The electrochemical surface area (ESCA) was used to know the active surface area of the catalyst, which was calculated from the double-layer capacitance (C_{dl}) obtained from the plot of the difference in cathodic and anodic sweep current density, $\Delta j = j_A - j_C$, versus scan

rates (v) from 10 to 50 mV/s, which yields a slope value equal to twice the C_{dl} as given in the equation (S3). [1]

$$\Delta j = v2C_{dl} \quad \text{...........(S3)}$$

The turnover frequency (TOF) is the critical parameter for studying the efficiency of each active site of the electrocatalyst. The number of moles of O2/H2 molecules that participated per unit of time can be measured from the equation (S4). [1]

$$\text{TOF} = \frac{IN_A}{Fn_1n_2}s^{-1} \quad \text{.........(S4)}$$

where I is current, N_A is the Avogadro constant, F is the Faraday constant, n_1 is the number of electrons transferred per molecule, and n_2 is the number of active sites. n_2 can be calculated from the equation (S5). [1]

$$n_2 = \frac{\frac{\frac{\int IV}{S}}{2}}{F} \quad \text{................(S5)}$$

∫IV/s is the absolute charge calculated by dividing the CV absolute area by the scan rate, S is the CV scan rate (50 mV/s), and F is the Faraday constant.

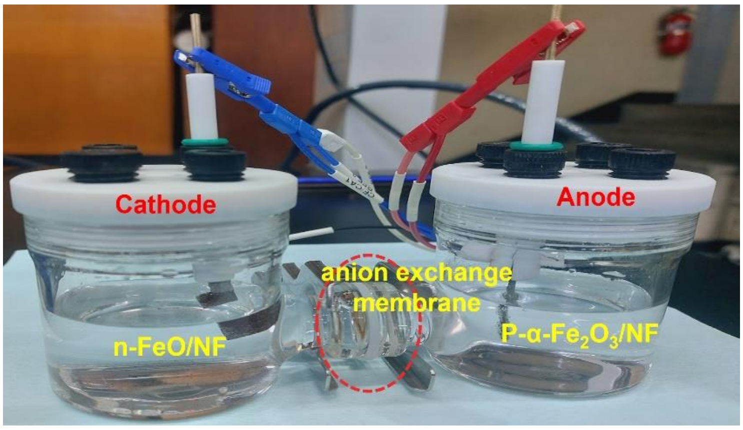

Scheme S1: H-cell Fabrication.

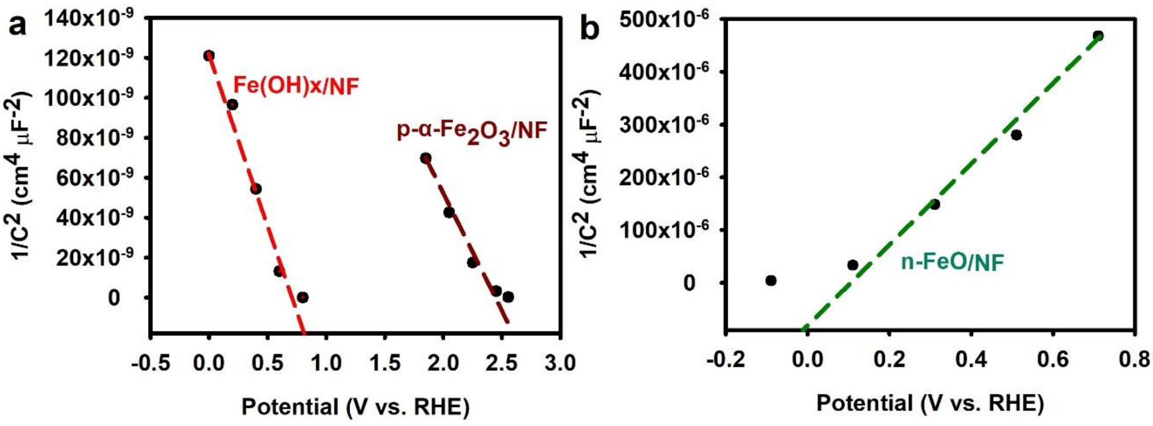

Figure S1. Mott-Schottky analysis for (**a**) p-α-Fe_2O_3/NF and $Fe(OH)_x$/NF and (**b**) n-FeO/NF electrodes.

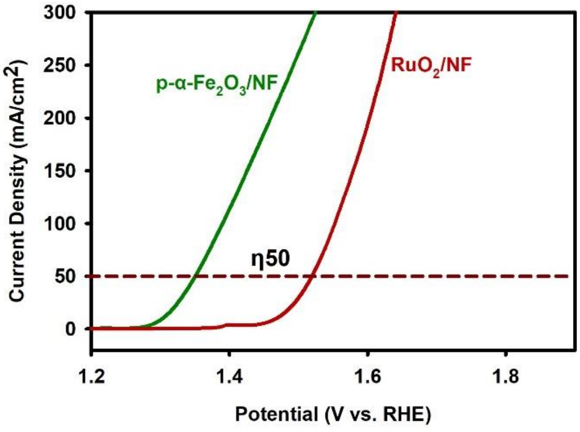

Figure S2. OER electrochemical studies for p-α-Fe_2O_3/NF with commercial RuO_2/NF LSV curve.

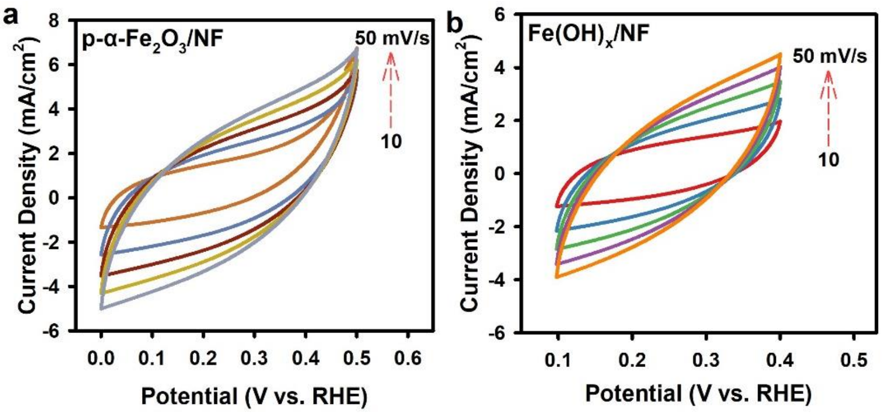

Figure S3. Double-layer capacitance measurements for determining the electrochemically active surface area for the (**a**) p-α-Fe_2O_3/NF and (**b**) $Fe(OH)_x$/NF electrodes in 1 M KOH, CV were measured in the non-faradaic region scan rates, varying from 10 to 50 mV/s.

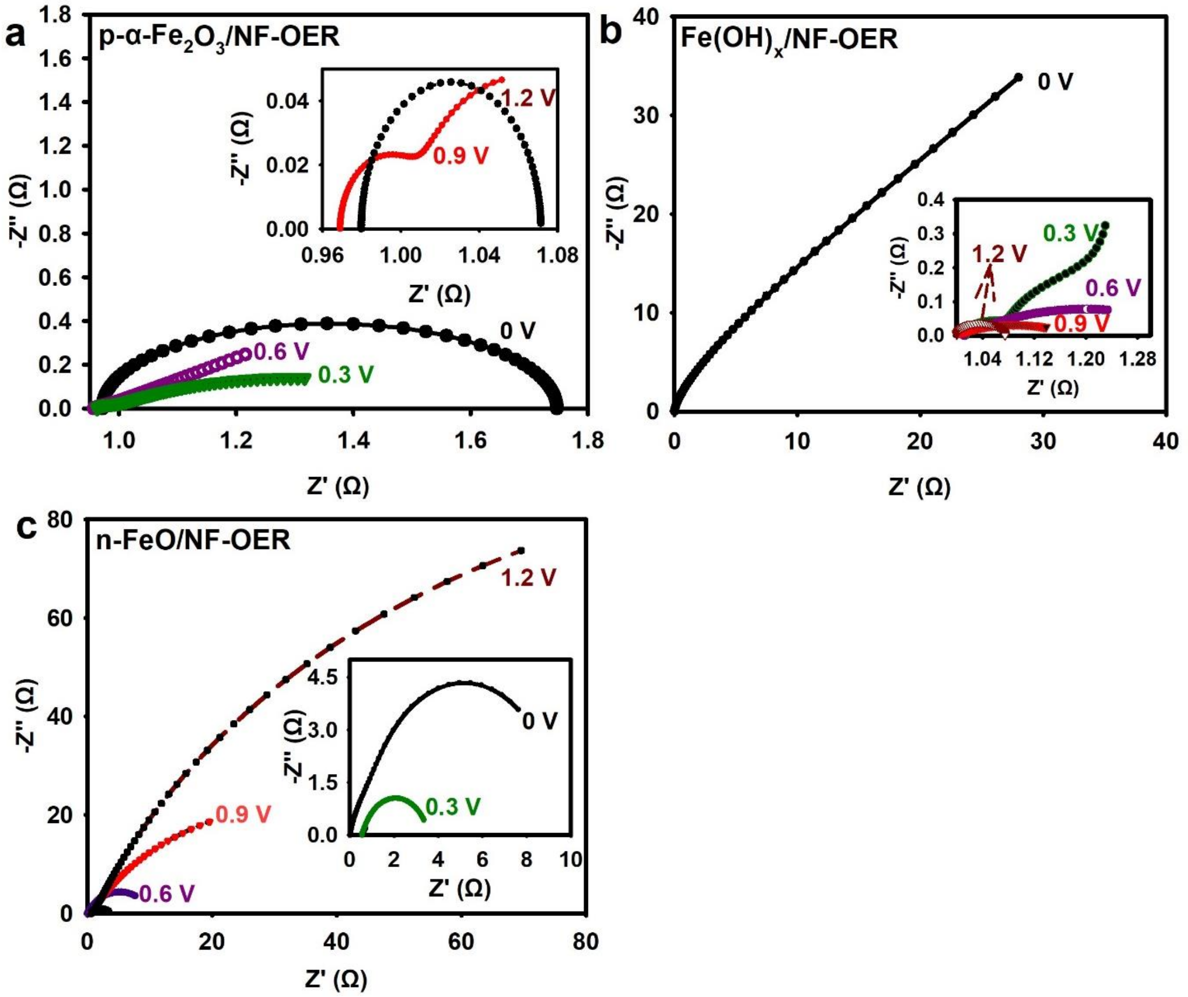

Figure S4: EIS measurement for (**a**) p-α-Fe_2O_3/NF, (**b**) $Fe(OH)_x$/NF, and (**c**) n-FeO/NF. All are performed by positive potential regions.

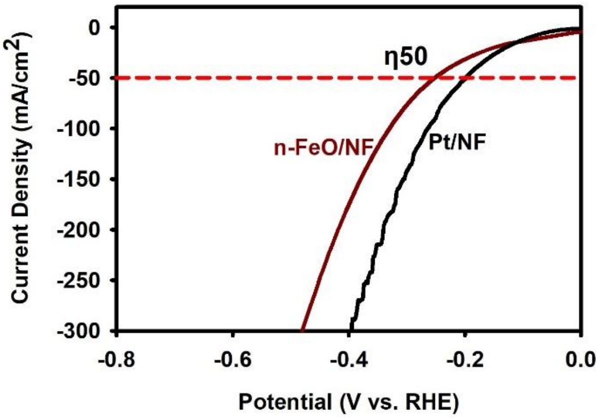

Figure S5. HER electrochemical studies for n-FeO/NF with commercial Pt/NF LSV curve.

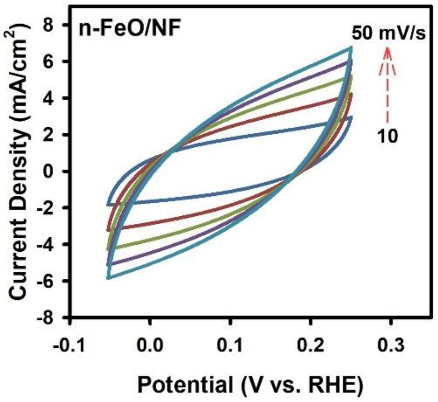

Figure S6. Double-layer capacitance measurements determined the electrochemically active surface area for the n-FeO/NF electrode in 1 M KOH. CV was measured in the non-faradaic region scan rates, varying from 10 to 50 mV/s.

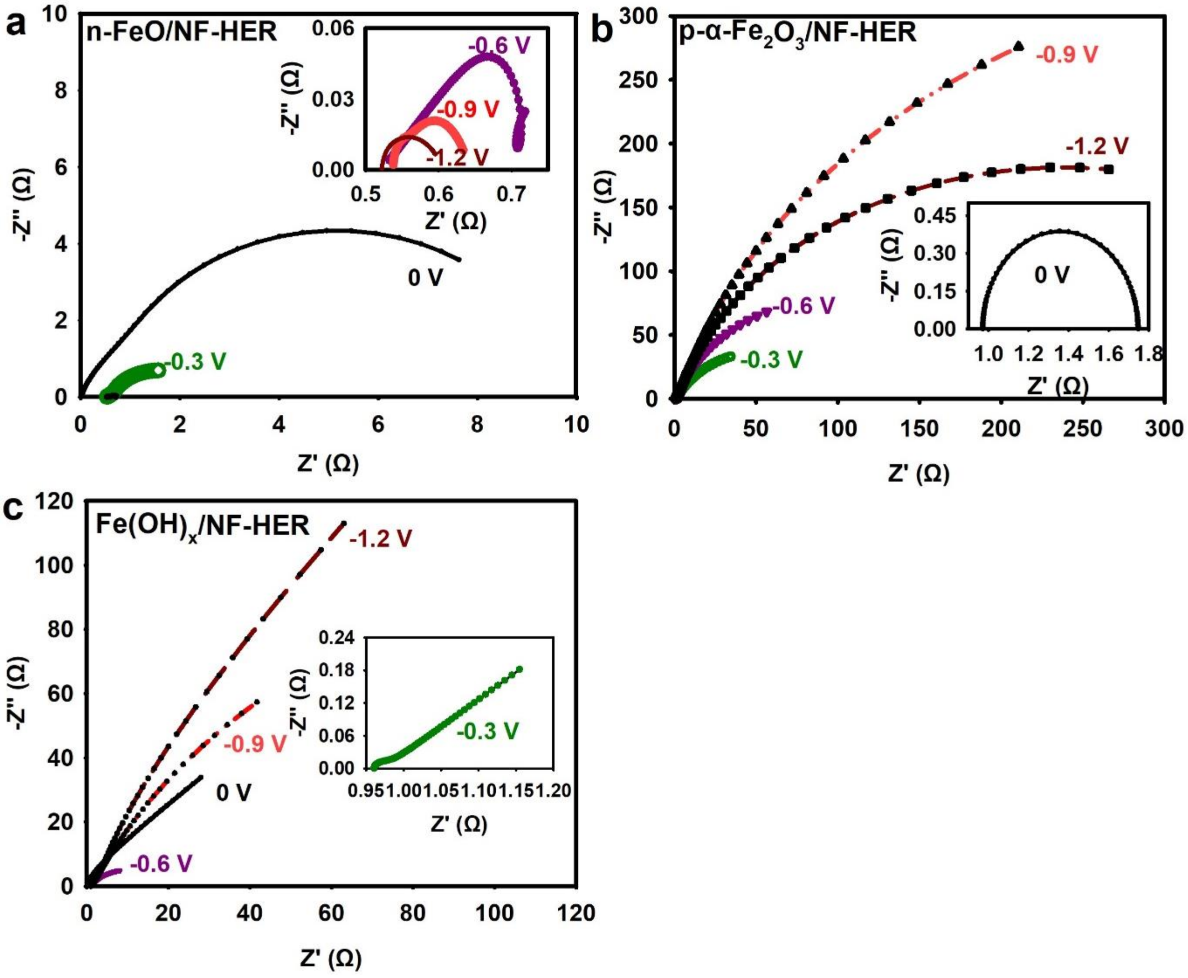

Figure S7: EIS measurement for (**a**) n-FeO/NF, (**b**) p-α-Fe_2O_3/NF, and (**c**) $Fe(OH)_x$/NF. All are performed by negative potential regions.

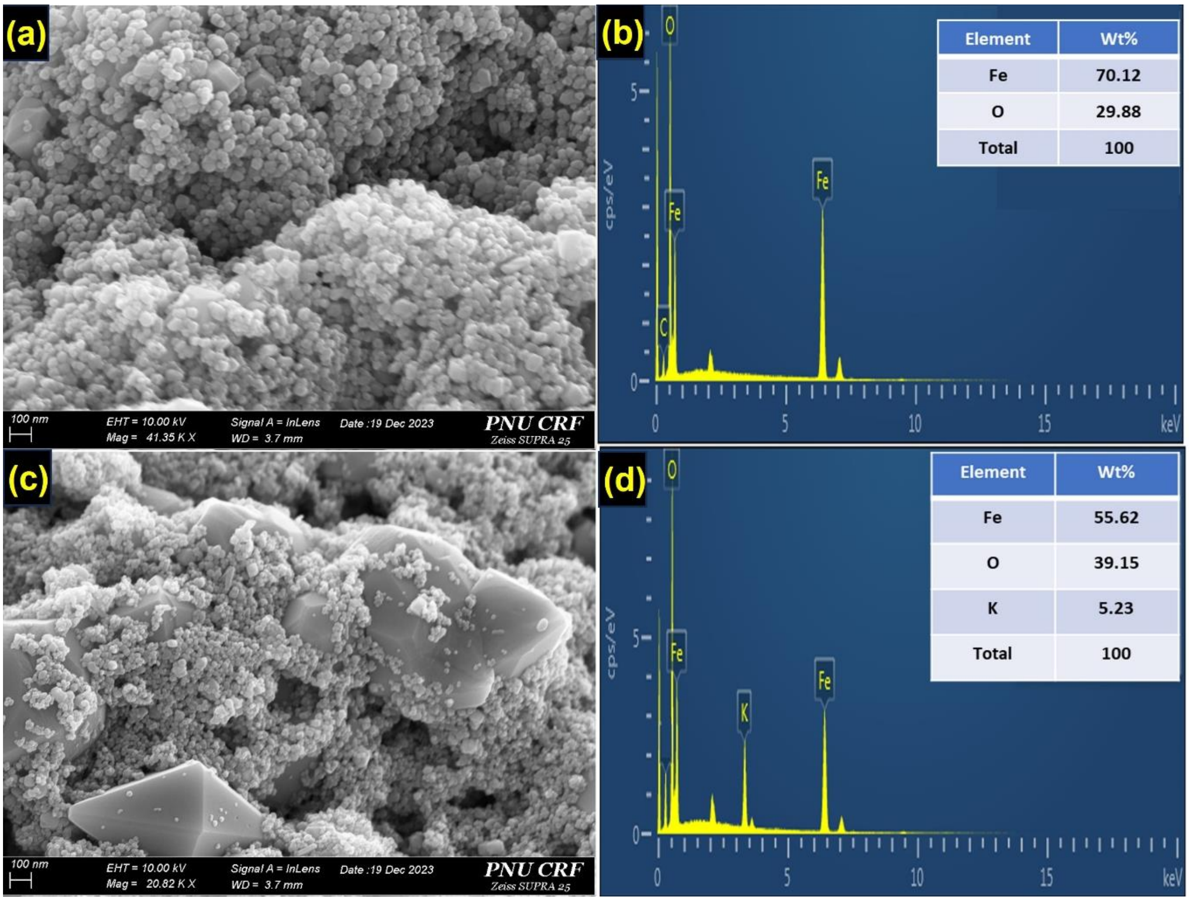

Figure S8: Post characterization FE-SEM and EDX analysis of n-FeO/NF electrode. (**a**,**b**) before stability, and (**c**,**d**) after stability.

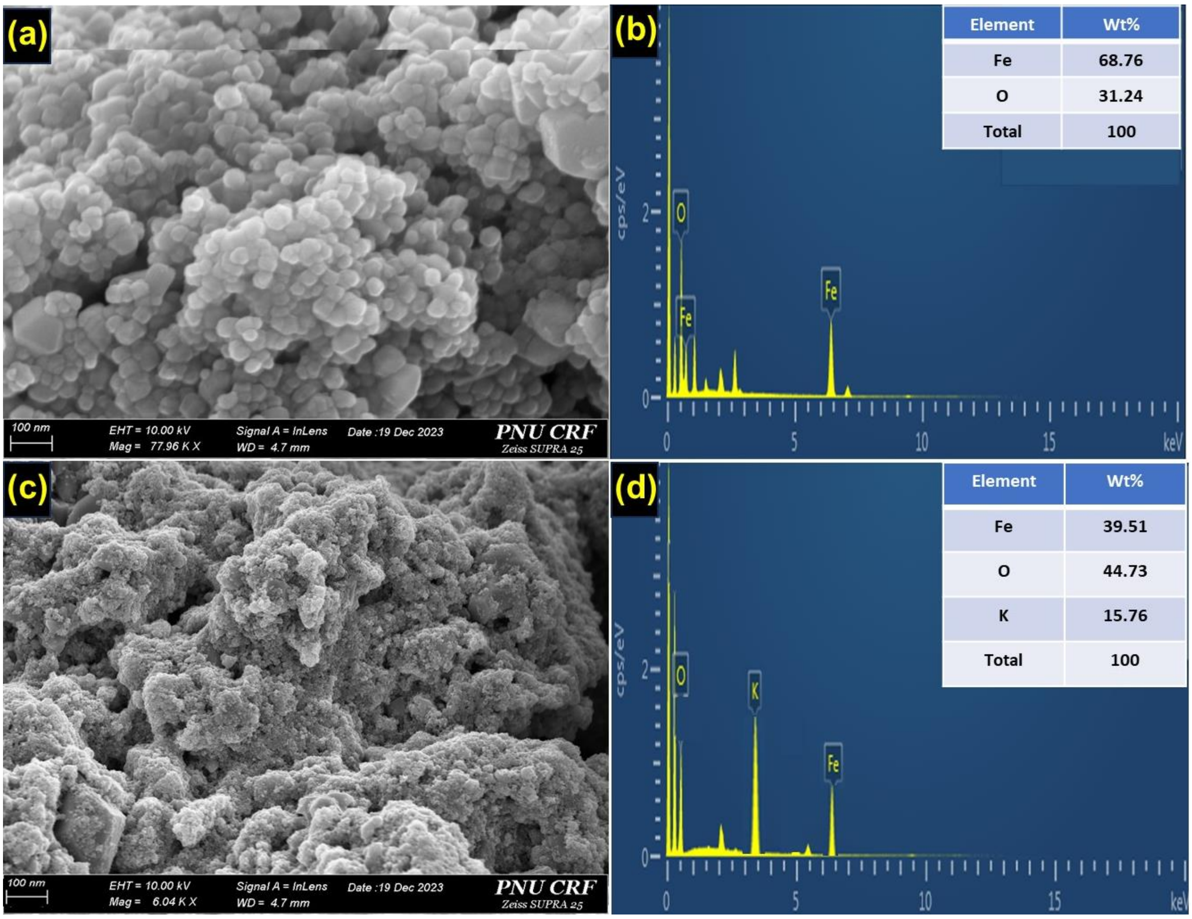

Figure S9: Post characterization FE-SEM and EDX analysis of p-α-Fe_2O_3/NF electrode. (**a**,**b**) before stability, and (**c**,**d**) after stability.

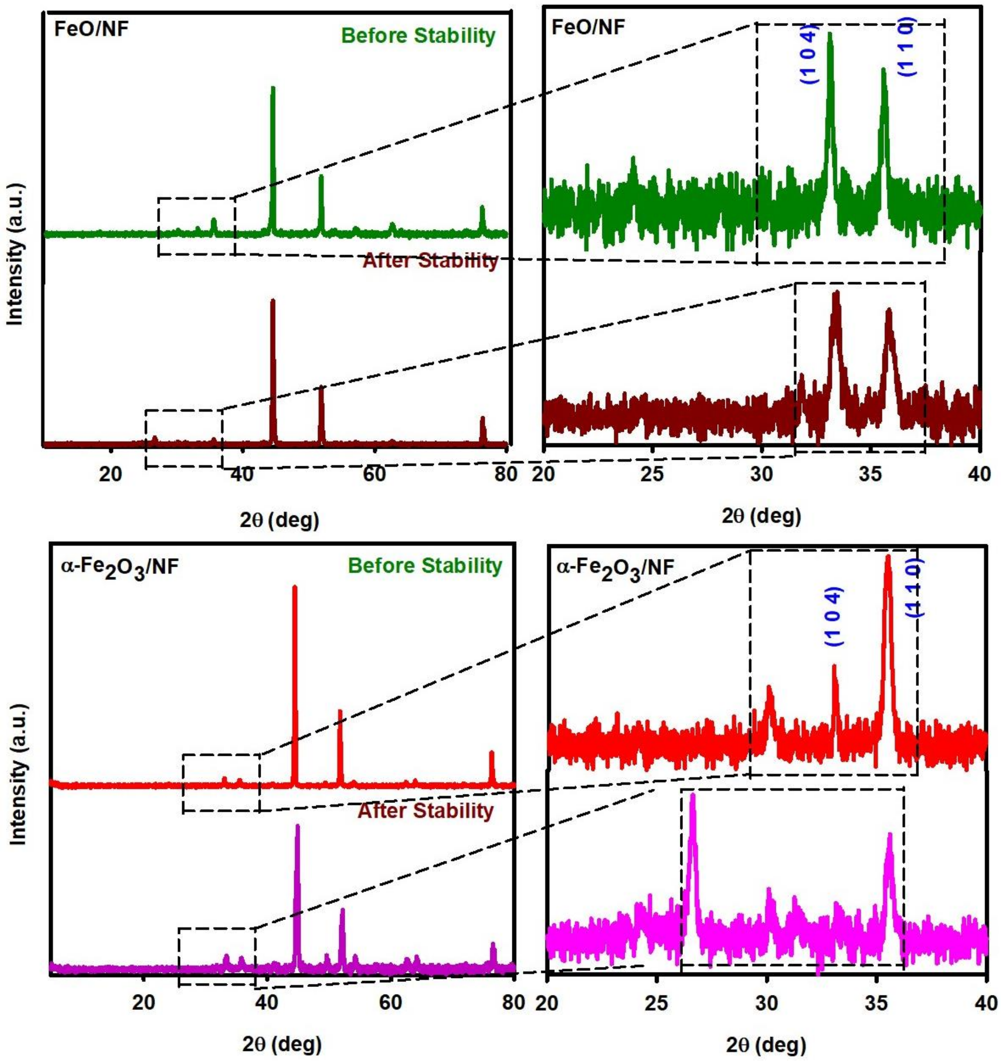

Figure S10: Post-characterization XRD analysis of (**a**,**b**) n-FeO/NF and (**c**,**d**) p-α-Fe_2O_3/NF electrodes.

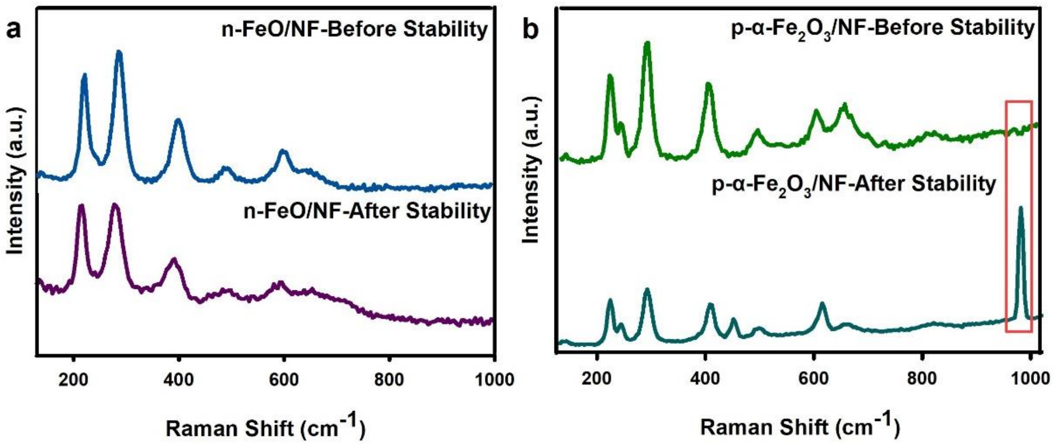

Figure S11: Post-characterization RAMAN analysis of (**a**) n-FeO/NF and (**b**) p-α-Fe_2O_3/NF electrodes.

Table S1: Iron Oxide NPs based catalysts for OER and HER activity

Catalyst	Electrolyte	OER η (mV)	HER η (mV)	Full-Cell (V)	Ref
Co_3O_4@100-FeO_x/CC	0.1 M KOH	314 @ 10 mA cm^{-1}	-	-	[2]
CN-FeO_x-OH	1 M KOH	322 @ 10 mA cm^{-1}	-	-	[3]
A-FeO_x	0.1 M KOH	427 @ 100 mA cm^{-1}	-	-	[4]
PdFe@FeO_x-C	1M KOH	-	320 mV @ 10 mA cm^{-1}	1.95 @ 10 mA cm^{-1}	[5]
NiCoFe@NiCoFeO NTAs/CFC	1M KOH	201 mV @ 10 mA cm^{-1}	-	-	[6]
FeO@$CuCo_2S_4$	1M KOH	-	107 mV @ 10 mA cm^{-1}	-	[7]
Ni-Fe_xO_x/ $FeNi_3$	1M KOH	267 @ 50 mA cm^{-1}	71 @ 50 mA cm^{-1}	1.58 @ 50 mA cm^{-1}	[8]
FeO_x/FeP	1M KOH	-	96 @ 10 mA cm^{-1}	-	[9]
n-FeO||p-α-Fe_2O_3	1M KOH	420 @ 50 mA cm^{-1}	250 @ 10 mA cm^{-1}	1.87 @ 50 mA cm^{-1}	**This Work**

Reference:

1. Manivelan, N.; Soundharrajan, V.; Kim, J.; Prabakar, K. Pentlandite Compound-Anchored CuSCN as a Stable Electrocatalyst in Highly Alkaline Solutions. ACS Sustain Chem Eng 2024, 12, 48–58.
2. Qingying, Z.; Guoyong, Yang.; Limin, T.; Hongwei, M.; Lingna, S.; Qianling, Z.; Libo, D.; Peixin, Z.; Xiangzhong, R.; Yongliang, Li. Enhanced Electrocatalytic Performance For Oxygen Evolution Reaction Via Active Interfaces Of Co_3O_4 Arrays@Feox/Carbon Cloth Heterostructure By Plasma-Enhanced Atomic Layer Deposition. Nanotechnology 2023, 34, 225703.

3. Tianyun, J.; Ning, Z.; Chaonan, Z.; Stefanos, M.; Zdenek, S.; Wei, L.; Pinjiang, L.; Tingting, L.; Yunpeng, Z.; Dewei, R. Improving C−N−FeOx Oxygen Evolution Electrocatalysts through hydroxyl-Modulated Local Coordination Environment. ACS Catal 2022, 12, 7443–7452.
4. Zhongbin, Z.; Stephen, A.G.; Glen, R.J.; Reza, A.; Xi, C.; Bo, W.; Dionisios, G. V.; Yushan, Y. Oxygen Evolution on Iron Oxide Nanoparticles: The Impact of Crystallinity and Size on the Overpotential. J. Electrochem. Soc 2021, 168, 034518.
5. Jordan, M.; Jaime, M.; Jose, L.S.; Juan, J.; Mohanad, D.D.; Guillermo, M.; Marcelo, E. D.; Pascual Ona, B. Bimetallic Intersection in PdFe@FeOx-C Nanomaterial for enhanced Water Splitting Electrocatalysis. Adv. Sustainable Syst 2022, 6, 2200096.
6. Yan, L.; Yiran, Y.; Linfeng, F.; Yi, L.; Qingzhao, H.; Guoge, Z.; Sin Yi, P.; Wei, L.; Chee Leung, M.; Xin, L.; Limin, Z.; Mingdeng, W.; Haitao, H. Valence Engineering via Selective Atomic Substitution on Tetrahedral Sites in Spinel Oxide for Highly Enhanced Oxygen Evolution Catalysis. J. Am. Chem. Soc 2019, 141, 8136–8145.
7. Abu Talha, A.A.; Abu Saad, A.; Pawar, S.M.; Bonggeun, S.; Hyungsang, K.; Hyunsik, I. Anti–corrosive FeO decorated $CuCo_2S_4$ as an efficient and durable electrocatalyst for hydrogen evolution reaction. Appl. Surf. Sci 2021, 539, 148229.
8. Abdul, Q.; Xiang, P.; Jianfa, Y.; Yuanduo, Q.; Jianhong, Z.; Zanling, H.; Hong, X.; Zhi, L.; Daniel, Q.T.; Paul, K.C.; Fushen, L.; Liangsheng, H. Highly Durable and Efficient Ni-FeO_x/$FeNi_3$ Electrocatalysts Synthesized by a Facile In Situ Combustion-Based Method for Overall Water Splitting with Large Current Densities. ACS Appl. Mater. Interfaces 2022, 14, 27842–27853.
9. Jianwen, H.; Ying, S.; Yadong, Z.; Wenqi, W.; Chunyang, W.; Yinghui, S.; Ruifeng, L.; Guifu, Z.; Yanrong, L.; Jie, X. FeO_x/FeP hybrid nanorods neutral hydrogen evolution electrocatalysis: insight into interface. J. Mater. Chem. A 2018, 6, 9467-9472.
